# Case of Ischuria Renalis

**Published:** 1831-01

**Authors:** J. Bird


### ISCHURIA RENALIS.

### *Case of Ischuria Renalis.* By J. Bird, Esq.*

Suppression of urine, from an affection of the kidneys, is so rare a disease as to meet but seldom the observation of medical men; and though a few cases are to be found scattered through different periodical publications, there is reason to think that it is neither well known nor generally understood. I remember hearing one of the most eminent surgeons in London relate, that, being called to a gentleman who had been attacked with apoplexy, preceded by suppression of urine, and on whom Dr. Maton was in attendance, he was surprised to hear Dr. Maton say that it was a case of Ischuria Renalis, as he was until then ignorant of the name and nature of the disease. Such being the fact, my professional brethren will receive this communication as an effort to extend our acquaintance with a malady which is obscure in its origin, and looked upon more as an original affection than as a symptom, which it generally is.

First day, 18th October, 1818. David James, an invalid of the garrison at Tannah, aged fifty years, came into hospital, complaining that he made urine in very small quantity; not more than a teacupful being passed in twenty-four hours. Had some nausea, but no fixed pain in any part. When first attacked, which was several hours previous to the time of his admission, he felt some uneasiness in the lumbar region, and was seized at the same time with vertigo; but having taken a draught of Tinct. Opii, with Spirit. Ether. Nitros., he felt easier. I ordered him a dose of Castor Oil; after the operation of which, the draught before given was to be repeated.

Second day. The oil had operated freely, but there was no alteration in the state of the symptoms. The draught was repeated several times in the course of the day.

Third day. The sensorium appeared much affected; as marked by a great inclination to sleep, an uncollected state of mind, and loss of memory. His eyes were of a suffused yellow colour, and were slightly injected with blood. The pulse was full and slow, and there was a convulsive motion in the arm. The catheter was introduced into the bladder, which contained no urine. He was bled to the extent of twenty ounces, and, after taking four grains of calomel, with an equal quantity of antimonial powder, had a blister applied to the lumbar region. The blood drawn was cupped, and threw up a little buff.

* Trans. Med. and Phys. Society, Calcutta.

Fourth day. There was less affection of the sensorium; but the urine did not increase in quantity. A brisk purgative of calomel and compound powder of jalap being given, its effect could not be accurately ascertained. A blister was also applied to the nape of the neck.

Fifth day. The affection of the sensorium increased: there was great inclination to sleep; and his tongue, which was brown, was covered by a horny crust. When told to show it, he did not withdraw it again until ordered to do so. Several strong purgative medicines were given without any effect.

Sixth day. The symptoms of coma continuing to increase, and his bowels remaining obstinately immoveable, purgative glysters were given very frequently, without any advantage.

Seventh day. The symptoms continued to gain ground, and death followed towards the afternoon.

*Dissection.* Abdomen: The stomach was much thickened; and its villous coat was unusually vascular. The liver, which was much enlarged, displaced the intestines, and had elongated the mesentery. The gall bladder contained a dark ropy bile, of the appearance of tar. The spleen, which was enlarged to more than five times its natural size, was firm and fleshy. The duodenum contained much brown mucus. The colon was contracted throughout its whole extent. The kidneys were enlarged to double their usual size, and were much altered in structure, having the pelves unnaturally small, and the mamillary processes so changed, as to be with difficulty distinguished from the generally diseased mass. In short, the impression left on the mind was, that urinary secretion had been performed very imperfectly for a long time previous to his death.

Head: A general effusion of serous fluid had taken place throughout the substance of the brain and into the lateral ventricles.

In cases similar to the preceding, vomiting and pain in the lumbar region have been observed to accompany the renal suppression of the urine; and the absence of these symptoms, in the present instance, can only be accounted for on the principle that a long residence in India, and intemperate habits, such as had operated in James's case, gradually exhaust the excitability of the constitution, and render it less sensibly alive to the impressions of disease. To this cause we must ascribe the exemption of the patient from febrile symptoms; and also the extreme slowness of his pulse, in an affection where the cupped appearance of the blood plainly indicated the existence of inflammatory action. This inflammation, in former instances, as ascertained by morbid anatomy, has been found in the kidneys, accompanied by enlargement of the liver; and the affection of the biliary organs, during life, was well marked in these instances, by symptoms characteristic of its existence. The generally diseased and enlarged state of the kidneys in the preceding case, could leave no doubt of a chronic inflammation having existed there; which was accompanied by diseased circulation and secretion of the liver.

In the case of Ischuria Renalis, described by Dr. Home in his Clinical Experiments, the patient had violent pain in his back and region of the liver; and, on dissection, there was found inflammation of the pylorus and duodenum accompanying the inflammation of the right kidney. The former is a very common appearance in cases of hepatitis producing death; and though Dr. Home says nothing about the liver, I cannot help thinking that, on minute examination, a deranged circulation of that organ would have been discovered to have existed. Another case, however, more decisively in favor of the opinion which I am anxious to impress on the profession, is to be found in vol. ii. of the Edinburgh Medical Essays, detailed by Mr. Balderston, where the patient could not lie on the left side; a symptom decisive of disease in the right lobe of the liver. After death, the liver was found very large, but not hard; and the right kidney of a monstrous size, and the blood-vessels on its surface very red and turgid.

From a knowledge of these facts, and constantly observing the very deficient and painful secretion of urine, in fever, and in most cases of hepatitis, with the extreme giddiness and languor, where there was congestion in the liver, I arrived at the conclusion, that Ischuria Renalis is more a general affection of the system, connected with diseased hepatic circulation, than a specific disease of the kidneys produced by inflammation. I am not prepared to deny, that there may be instances of this disease being original and uncomplicated; but in those where apoplexy and coma follow in its train, the liver and portal circle should, I think, occupy particular attention in the treatment.

Plethora, spasms, worms, mucus, and paralytic affection, have been each assigned as causes of Ischuria; and it has received as many different names accordingly. One or all these may be occasionally observed to influence this disease; which has its origin, however, more immediately from an increasing congestion in the liver, and other abdominal viscera; with a change, perhaps, in the elementary principles of the urine, and a temporary loss of excitability in the constitution.

Would it not, therefore, be advisable, after bleeding the patient, while immersed in a warm bath, and applying a blister to his loins, to administer calomel in ten grain doses, with such diuretics as determine to the skin, viz. Camphor mixture, and Spirit. Ether. Nitros. until soreness of the mouth followed? This certainly is the plan I would pursue in future, and should recommend that the bowels be relieved by purging clysters. If no increased secretion of urine followed the purgative effect of the calomel, within twenty or thirty hours, the medicine should be repeated in a large dose, until its constitutional effect be obtained; and from my observation of this disease, I should anticipate a favorable result. Purging will undoubtedly be beneficial, but I should not be disposed to trust to that entirely; as spontaneous diarrhœa has accompanied cases of Ischuria Renalis without being of benefit. All the forms of opium ought, I think, to be excluded from the treatment, on account of the diseased secretion from the liver.

---

